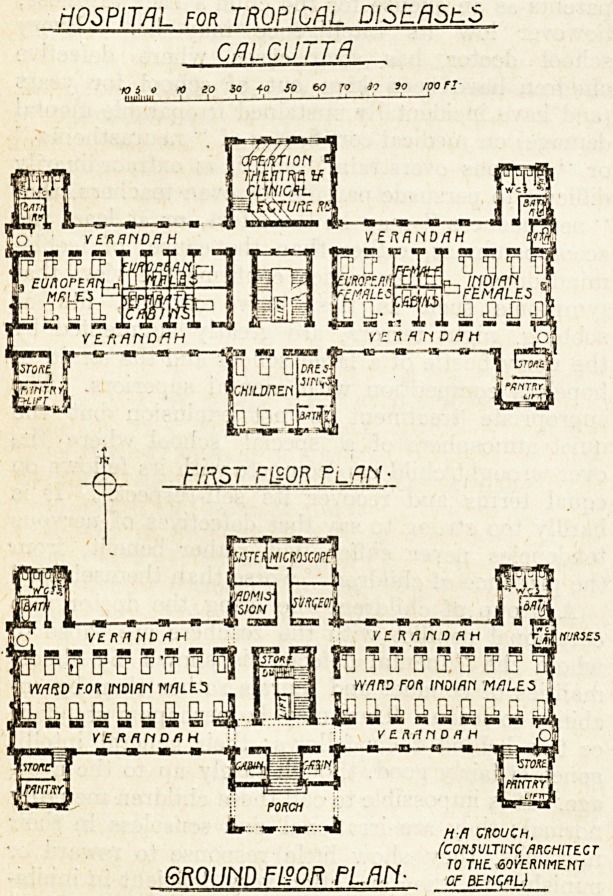# Hospital for Tropical Diseases, Calcutta

**Published:** 1916-06-10

**Authors:** 


### HOSPITAL ARCHITECTURE AND CONSTRUCTION.

### Hospital for Tropical Diseases, Calcutta.

This hospital, when completed, will consist of a basement and three floors. Quarters are provided in the central block of the top floor for a resident medical officer and a sister in charge, and on the roof in turrets for two Indian house physicians. Kitchens are provided in the basement. The two floors will accommodate eighty patients, and include male and female wards for both Europeans and Indians, and a children's ward over the entrance porch.

Ground-floor Plan.

It will be noticed on reference to the ground-floor plan that two large wards containing eighteen beds each are provided for Indian male patients and are separated by a central block containing entrance hall, staircase, and cross corridors from which the wards are entered. A passenger lift is placed in the well of the staircase and serves each floor. Leading off the main corridor and on either side of the entrance are two cabins containing one bed each.

This central portion extends north and has four well-arranged rooms—viz., admission room, surgeons' room, sisters' room, and a clinical microscopical room. Running practically the whole length of the building on the north and south fronts are wide verandahs, and on the south front, at the extreme ends, and forming wings, is a store-room and pantry, a lift serving each floor being provided in the latter. Similar wings are placed on the north side forming sanitary offices, consisting of bathroom, opening directly off verandah, and three water-closets and sink-room.

At the east end of this verandah is a lavatory for nurses. These sanitary offices, we consider, should have had cut-off lobbies, and the placing of the nurses' lavatory at the end of the verandah is an unfortunate piece of planning.

First-floor Plan.

This floor contains, in the west wing, a ward for nine European males and a series of single-bed cabins. In the east wing are two wards, one for nine Indian females and one for five Indian females; between these are placed four single-bed cabins. In the north portion of the central wing is an operation theatre and clinical lecture room with a gallery. The south portion of this wing contains a ward for six children, bathroom, and water-closet. Opening off the landing is a room for dressings.

The arrangement of stores, pantries, and sanitary blocks is similar to those on the ground floor, the only exception being an extra bathroom in the east wing.

Generally speaking the plan is good, and we consider the building will be an excellent one for its purpose. The design is by Mr. H. A. Crouch, consulting architect to the Government of Bengal.

## Figures and Tables

**Figure f1:**